# Fully-biobased UV-absorbing nanoparticles from ethyl cellulose and zein for environmentally friendly photoprotection†

**DOI:** 10.1039/c8ra02674b

**Published:** 2018-07-12

**Authors:** Douglas R. Hayden, Heleen V. M. Kibbelaar, Arnout Imhof, Krassimir P. Velikov

**Affiliations:** Soft Condensed Matter, Debye Institute for Nanomaterials Science, Utrecht University Princetonplein 1 3584 CC Utrecht The Netherlands d.r.hayden@uu.nl a.imhof@uu.nl; Unilever R&D Vlaardingen Olivier van Noortlaan 120 3133 AT Vlaardingen The Netherlands; Institute of Physics, University of Amsterdam Science Park 904 1098 XH Amsterdam The Netherlands

## Abstract

Effective photoprotection is a vital consumer issue. However, there are many concerns regarding the adverse environmental and health impacts associated with current organic and inorganic UV filters. Here, we prepare fully-biobased UV-absorbing nanoparticles from ethyl cellulose (ECNPs) and zein (ZNPs) with encapsulated biobased photoprotectants obtainable from plants and foods (quercetin, retinol, and *p*-coumaric acid), which have the potential to satisfy both environmental and health issues in photoprotection. We show the ability of ECNPs and ZNPs to be easily tuned compositionally to obtain uniform, broadband UV spectrum absorbance profiles, and prepare transparent UV-absorbing coatings from the ECNPs. We find that the maximum loadings for retinol, quercetin, and *p*-coumaric acid into the ECNPs are 31 wt%, 14 wt%, and 13 wt% respectively. The ECNP size remains constant (except for the largest loading of retinol, 31 wt%) and the absolute zeta potential increases upon increasing the loading of quercetin and retinol, whereas increasing the loading of *p*-coumaric acid results in increasing the particle size and a lower absolute zeta potential. We find that quercetin and retinol are effectively retained inside the ECNPs at 64–70% after 72 hours. These results have significant implications for the development of novel photoprotection technologies and functional nanoparticles.

## Abstract 2

UV-absorbing nanoparticles are prepared with an entirely biobased composition, as a novel environmentally-friendly photoprotection technology.

## Graphical abstract

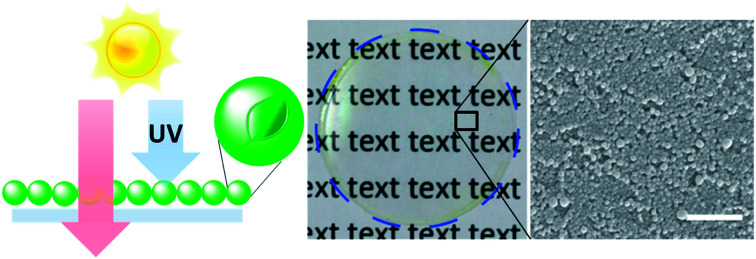

## Introduction

Excessive exposure to UV radiation from sunlight can lead to the degradation of foods and packaging materials, as well as multiple adverse health effects such as sunburn, accelerated skin aging, and the vast majority of skin cancers.^1,2^ Protection against UV radiation *via* the use of sunscreens is therefore vital for many consumer products and crucial for human health.

Many current cosmetic sunscreens provide protection across the entire UV spectrum (*λ* = 290–380 nm) *via* the use of multiple synthetic organic and inorganic UV filters. Despite the effectiveness of synthetic organic and inorganic UV filters in protecting against UV radiation, their use in large quantities has been reported to have significant adverse environmental and health effects. For example, large concentrations of synthetic organic UV filters in coastal recreational areas have been strongly linked with accelerated damage to coral reefs^3,4^ and marine phytoplankton^5^ by promoting viral infections, which has significant implications for the local ecosystem. Inorganic UV filters such as TiO_2_ nanoparticles have also been reported as potentially toxic to marine life because they remain photocatalytically active in the environment.^6,7^ As for health concerns, inorganic and synthetic organic UV filters have been identified as phototoxic,^8,9^ potent skin allergens,^10^ and have been reported to penetrate the skin and act as endocrine disruptors in the bloodstream.^11,12^

One method to address the health concerns of inorganic and synthetic organic UV filters is to minimize skin contact with the UV filters. Skin contact can be minimized for inorganic UV filters *via* coating the particles (*i.e.* with silica/alumina) and for synthetic organic UV filters *via* encapsulation into nanoparticles from materials such as silica,^13^ gelatin,^14^ lipids,^15^ poly-lactide,^16^ and ethyl cellulose (EC).^9^ The coating of inorganic UV filters and encapsulation of synthetic organic UV filters can result in significant reduction of direct skin contact,^12,17^ skin penetration,^12^ and improvements in the phototoxicity.^9,13^ Furthermore, encapsulation of synthetic organic UV filters into nanoparticles also reduces the need for unnecessary chemicals (*i.e.* surfactants used in formulation), can increase photostability,^16^ and potentially allows higher loadings in formulations – the amount of UV filter in the formulation is no longer limited by its solubility.^18^ Despite the advantages of nanoencapsulation, the adverse health effects of inorganic and synthetic organic UV filters cannot be completely eliminated and their environmental impact remains a concern. Thus, the replacement of these UV filters with eco-friendly, natural, biobased photoprotectants – which are potentially safer^19^ – and encapsulation into biocompatible eco-friendly nanoparticles has the potential to satisfy both environmental and health concerns whilst providing effective photoprotection.

There are many known biobased photoprotectants, such as flavonoids (*i.e.* quercetin and rutin), lignin (*i.e. p*-coumaric acid), and carotenoids (*i.e.* β-carotene, lutein, lycopene, retinol). Quercetin and rutin are the most extensively studied biobased photoprotectants, and both have even been individually incorporated into solid-lipid nanoparticles (SLNs) for sunscreen applications.^14,19,20^ Despite this, there is still a need to develop UV-absorbing nanoparticles with an entirely natural composition which can: (i) efficiently encapsulate multiple biobased photoprotectants, (ii) provide broadband and uniform UV absorbance, (iii) prepare effective UV-protective coatings, and (iv) be potentially used for multiple solvent systems *e.g.* oil and emulsion (SLNs may dissolve in oil continuous formulations), for effective eco-friendly photoprotection. Here, we develop fully-biobased UV-absorbing nanoparticles *via* the encapsulation of multiple biobased photoprotectants together into biobased ethyl cellulose nanoparticles (ECNPs) and biobased zein nanoparticles (ZNPs) using an upscalable technique. As a protein, zein may not be suitable for skincare applications but is potentially very interesting for edible photoprotection applications such as food coatings, and is moreover an interesting material because it absorbs UV light itself primarily due to the presence of xanthophylls and β-carotene.^21^ We show that the fully-biobased UV-absorbing ECNPs and ZNPs with encapsulated quercetin, retinol, and *p*-coumaric acid can provide effective uniform broadband UV spectrum protection. We then focus on the UV-absorbing ECNPs and prepare transparent, flexible coatings with tunable thicknesses and study the photodegradation of these coatings. Additionally, we study the retention of the biobased photoprotectants inside the ECNPs and study the incorporation of the biobased photoprotectants into the ECNPs.

These findings are significant for the development of safer and more environmentally friendly methods of photoprotection, in applications such as cosmetic sunscreen formulations, packaging materials and food coatings. Furthermore, these findings also have important implications for the more general fields of UV protective coatings and functional nanoparticles.

## Experimental

### Materials

Ethyl cellulose (100 cP, lot number MKBT0521V), zein (lot number SLBL9380V), quercetin (≥95%, water solubility 60 mg L^−1^ at 16 °C (DrugBank Database)), retinol (≥95%, water solubility 0.67 mg L^−1^ at 25 °C (DrugBank database)), and *p*-coumaric acid (98%, water solubility 1.02 mg L^−1^ at 24 °C (DrugBank database)), were all purchased from Sigma Aldrich. Ethanol (100%) was purchased from Interchema and pure water was used from a Millipore system.

### ECNPs/ZNPs with biobased photoprotectants encapsulated

ECNPs and ZNPs with quercetin, retinol and *p*-coumaric acid were prepared *via* an antisolvent precipitation technique. Briefly, 0.275 g of EC (for ECNPs) or 0.35 g zein (for ZNPs) and 7 wt% quercetin (thus 0.020 g for ECNPs and 0.025 g for ZNPs), 1.5 wt% *p*-coumaric acid and 1.5 wt% retinol (0.004 g for ECNPs and 0.005 g for ZNPs) were dissolved together in 50 mL ethanol (for ECNPs) or 50 mL of an ethanol/water mixture (80 (v/v)% EtOH) (for ZNPs). This solution was then poured into water (150 mL) under fast magnetic stirring, resulting in the spontaneous formation of ECNPs/ZNPs with encapsulated UV filters. Rotary evaporation removed ethanol and some water to give a 50 mL aqueous dispersion of ECNPs or ZNPs with encapsulated UV filters. The ECNP/ZNP dispersions were filtered through filter paper to remove any large aggregates formed during the antisolvent precipitation. The particles were characterised by Scanning Electron Microscopy (SEM, FEI XL30FEG, samples were sputter coated with platinum), spectrophotometry (HP 8452a), Dynamic Light Scattering (DLS) and zeta potential measurements (Malvern Zetasizer, particle size distributions obtained using a CONTIN fitting and zeta potential measurements performed in presence of 10 mM NaCl background salt one day after preparation of the particles). DLS and zeta potential measurements were performed in triplicate.

### Preparation of UV-protective coatings and photodegradation studies

Coatings were prepared by spin coating (SCS P6700) a concentrated dispersion (30 g L^−1^) of the fully-biobased UV-absorbing ECNPs onto plasma-cleaned, circular glass microscope cover slips at 1800 rpm for 1 minute. For photodegradation studies, the coating was subjected to irradiation by a 75 W xenon lamp at a distance of 20 cm (a flux of 3 mW cm^−2^ between 300–400 nm). The absorbance was measured hourly for four hours. Coating thickness was measured by SEM imaging (Fig. S5†).

### Determination of the maximum loadings of photoprotectants into ECNPs

Loadings of photoprotectant inside ECNPs were determined by a spectroscopic method. Briefly, the absorbance of a known concentration of aqueous ECNPs containing encapsulated photoprotectant at the peak of the spectrum was compared with a calibration curve prepared from a series of known concentrations of photoprotectant dissolved in ethanol. Here we make the assumption that the contribution of particle scattering to absorbance is negligible, which is supported by Fig. S9.† Particle dispersions were prepared in duplicate to give the error bars in Fig. 3a.

### Retention of photoprotectants inside ECNPs

The retention of the photoprotectants inside the ECNPs was measured as follows: 10 mL of ECNPs with one biobased photoprotectant encapsulated (at 3 wt% for retinol and 4 wt% for quercetin, dispersion concentrations both 5 g L^−1^) in dialysis membrane tubing (pore size 40 kDa) was placed into 300 mL of water at room temperature under gentle magnetic stirring. Retention was determined by measuring the absorbance of the contents of the dialysis tubing after 24 h, 36 h, and 72 h, in separate experiments to avoid inaccuracy through measurement.

## Results and discussion

### Preparation of fully-biobased UV-absorbing nanoparticles from EC and zein

**Chart 1:**
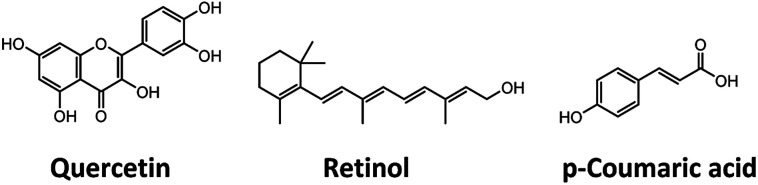
Molecular structures of the biobased photoprotectants: quercetin, retinol, and *p*-coumaric acid.

We investigated the incorporation of three biobased photoprotectants quercetin, retinol, and *p*-coumaric acid (chemical structures in Chart 1) into ECNPs and ZNPs. The biobased photoprotectants were specifically chosen because of their solubility properties (hydrophobic) and because together they absorb across the entire UV spectrum (290–380 nm) – broadband UV spectrum protection is of paramount importance for sunscreen applications. The three photoprotectants were incorporated into ECNPs and ZNPs (<100 nm) *via* a simple, upscalable, surfactant-free “antisolvent precipitation” technique.^9,22^ The antisolvent precipitation procedure relies on the co-precipitation of the hydrophobic photoprotectants together with the hydrophobic EC or zein.

**Fig. 1:**
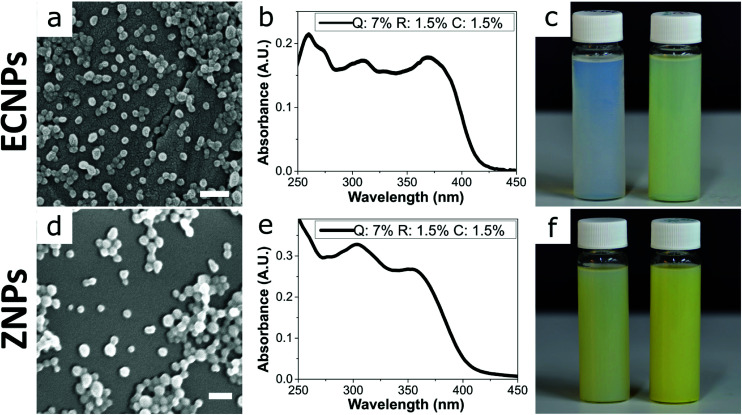
(a and d) SEM images of (a) ECNPs and (d) ZNPs with encapsulated biobased photoprotectants: quercetin (Q), retinol (R), and *p*-coumaric acid (C). Scale bar 200 nm. (b and e) Absorbance measurements of the (b) ECNPs and (e) ZNPs with encapsulated UV filters showing a broadband absorbance profile across the UV spectrum. Both dispersions were measured by spectrophotometry at equal concentration (5 × 10^−2^ g L^−1^). (c and f) Photo of the (c) ECNP and (f) ZNP dispersions without (left) and with (right) encapsulated photoprotectants.

By using this procedure, we prepared fully-biobased ECNPs and ZNPs with encapsulated quercetin, retinol, and *p*-coumaric acid. Both the prepared ECNPs and ZNPs with encapsulated biobased photoprotectants were roughly spherical and had an average size of 70–76 nm, as seen by dynamic light scattering (DLS) measurements and SEM imaging (Fig. 1a and d and DLS measurements Fig S1†). We found that we could tune the amount of each biobased photoprotectant encapsulated simply by varying the ratio of each photoprotectant initially dissolved in the ethanol or ethanol/water solution before undergoing the antisolvent precipitation. Therefore, we always added the same total amount of the three biobased photoprotectants (10 wt% of the amount of EC/zein). We always kept the total loading below 10 wt% because ECNPs are known to encapsulate organic UV filters efficiently to this weight percentage,^23^ and we wanted to stay under this value so that all photoprotectants were efficiently encapsulated and therefore the absorbance profiles from the ECNPs were reproducible (Fig. S3† shows unpredictability of the ECNP absorbance profile upon loadings greater than 10 wt%). We found that we could achieve a uniform absorbance profile across the entire UV spectrum for both ECNPs and ZNPs with encapsulated quercetin, *p*-coumaric acid and retinol in a ratio of 7 : 1.5 : 1.5 (Fig. 1b and e), whereas a 1 : 1 : 1 ratio resulted in greater absorbance in the UVB region of the spectrum (Fig. S2b†). Interestingly, the absorbance of zein itself contributed slightly to the overall absorbance of the ZNPs (*cf.*Fig. 1b and e), in which the absorbance profile remained approximately uniform but slightly greater in the UVB region and also with some absorbance at wavelengths greater than 425 nm. The absorbance of the ZNPs at higher wavelengths resulted in a distinctive yellow colour of the ZNPs^24^ compared to the ECNPs^25^ (Fig. 1c left vial *vs.*Fig. 1f left vial). The encapsulation of quercetin, which was necessary to achieve absorbance in the UVA region of the spectrum, also gave the dispersions a yellow colour due to its absorbance in the blue region of the visible light spectrum.^19^ Therefore, the resultant ECNP and ZNP dispersions both had a yellow colour (Fig. 1c right vial and Fig. 1f right vial), unlike UV-absorbing ECNPs prepared with synthetic UV filters encapsulated which can provide uniform absorbance across the entire UV spectrum without a yellow colour because synthetic UVA filters have been developed which do not absorb into the visible spectrum.^9^ We found that the ECNPs had a zeta potential of −25 mV (at the pH of MilliQ water, pH = 5–6), the ZNPs had a zeta potential of +30 mV (at pH = 3.5) and both were colloidally stable at these respective pH values.

### Preparation of fully-biobased, transparent, UV-absorbing coatings from the UV-absorbing ECNPs

We then focused on the ECNPs because of their superior colour and ease of handling with regards to their stability at close-to neutral pH – zein has an isoelectric point at pH 6.8.^22^ The ability for these UV-absorbing nanoparticles to form coatings is essential for photoprotection applications such as cosmetic sunscreens and packaging materials.

**Fig. 2:**
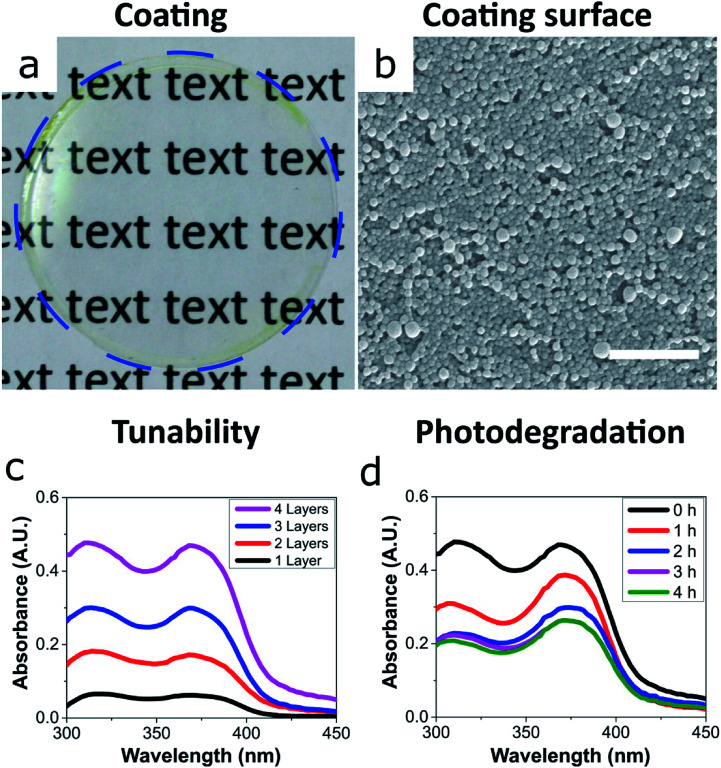
(a) Photo of a transparent and uniform coating of the ECNPs on a glass cover slip. A blue dashed line indicates the outside edge of the glass cover slip. (b) SEM image of the coating surface, scale bar 1 μm. (c) Absorbance measurements of the ECNP coating showing the absorbance of each successive spin coated layer. Wavelengths lower than *λ* = 300 nm are not shown as they are absorbed by the glass coverslip. (d) Absorbance measurements showing the degradation of the absorbance of the 4-layer coating of ECNPs when irritated by artificial sunlight for 4 h.

We prepared uniform and transparent coatings from the fully-biobased UV-absorbing ECNPs (Fig. 2a and b), with uniform absorbance profiles across the entire UV spectrum, *via* spin coating. We could completely tune the coating absorbance simply *via* the spin coating of additional layers on top of the original (Fig. 2c), and we found that a 4-layer coating had a thickness of 373 ± 17 nm (Fig. S5†). Despite the noticeable yellow colour of the ECNP dispersions, the coating appeared very transparent and a yellow tinge is only visible upon close inspection of the coating at the edges (Fig. 2a), due to the well-known “coffee-ring” drying effect where higher concentrations of particles end up at the edges in coatings prepared by spin coating.^26^ The 373 ± 17 nm fully-biobased coating therefore shows effective and uniform absorbance across the entire UV spectrum although, interestingly, does demonstrate a lower absorbance (average 0.44 A.U. between *λ* = 290–380 nm) than a similarly thick (∼312 nm) coating of similarly-sized ECNPs with a similar loading of synthetic UV filters of ∼10 wt% (average coating absorbance 0.81 A.U. between *λ* = 290–380 nm).^9^

We also investigated the photostability of the coating – photostability is essential if coatings are to provide effective photoprotection. Moreover, the photostability is known to vary greatly between commercialised UV filters.^2,27,28^ In order to investigate the photostability of the fully-biobased UV-absorbing ECNPs, the coating was irradiated by artificial sunlight for 4 hours and the coating absorbance was measured at hourly intervals (Fig. 2d). The coating was subjected to a total flux of 432 kJ m^−2^ UV irradiation (between *λ* = 300–400 nm) over the 4 hours which translates to 2 hours and 24 minutes of summer sunlight in Nice (France) at noon.^29^ We found that the morphology of the ECNPs on the coating surface remained identical after this dose of UV irradiation (Fig. S6†). However, the coating absorbance degraded by approximately 50% after the total of 4 hours of irradiation. The absorbance loss can be attributed to the degradation of the plant-based photoprotectants encapsulated inside the ECNPs, which are known to lose absorbance upon UV irradiation.^27,30,31^ This coating degradation is about two times as large as what is reported for ECNPs with encapsulated synthetic organic UV filters.^9^ Interestingly, the coating absorbance begins to degrade very quickly, especially in the UVB region – but this degradation then plateaus after about 2 hours of irradiation. This is likely due to the photoisomerisation of retinol into a mixture of isomers^27^ as well as the *cis*–*trans* isomerisation of *p*-coumaric acid,^30^ which both result in absorbance degradation in the UVB region. Overall, the coating maintains effective uniform absorbance across the entire UV spectrum upon irradiation by artificial sunlight and, therefore, these fully-biobased UV-absorbing ECNPs can prepare effective UV protective coatings with photodegradation values comparable with synthetic UV filters.

### Investigation into the encapsulation of biobased photoprotectants into ECNPs

We then investigated the encapsulation of the individual biobased photoprotectants into the ECNPs. Studying the encapsulation of each of these photoprotectants into the ECNPs is crucial in giving us a better understanding of how to prepare effective fully-biobased UV-absorbing NPs. Here, we studied two phenomena: (i) the maximum loadings of the biobased photoprotectants into the ECNPs, and (ii) the effect of increasing loadings on the size and stability of the ECNPs. It is important that the ECNPs can efficiently encapsulate large amounts of the biobased photoprotectants whilst remaining colloidally stable and without significant changes to the morphology of the particles – preferably the ECNPs will remain small (<100 nm) to minimize scattering of visible light when applied as a coating and therefore remain attractive for cosmetic applications.

**Fig. 3:**
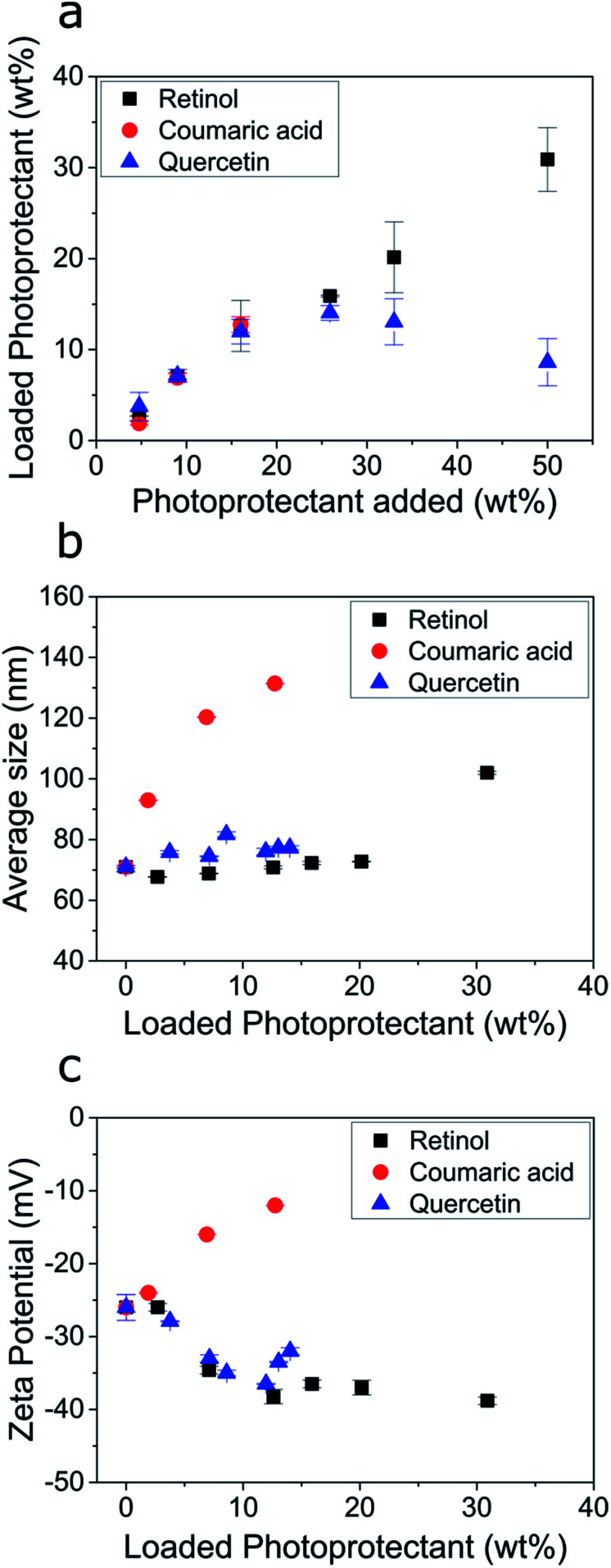
(a) Amount of photoprotectant loaded into the ECNPs (loaded photoprotectant) as a function of the amount of photoprotectant added to the synthesis (specifically: the amount dissolved in the ethanol phase before undergoing the antisolvent precipitation). (b) The average ECNP size measurements as determined by DLS as a function of the photoprotectant loading. (c) The zeta potential measurements of the ECNP dispersions as a function of the photoprotectant loading. The raw data for (a–c) is shown in Table S1.†

We found that the biobased photoprotectants could all be efficiently encapsulated into the ECNPs (Fig. 3a), but the extent of loading varied between photoprotectants. For example, 14 wt% quercetin and 13 wt% *p*-coumaric acid could be encapsulated into the ECNPs, whereas up to 31 wt% for retinol (Fig. 3a). Addition of larger amounts of quercetin to the synthesis simply resulted in similar particle loading for the ECNPs (12–14 wt%, Fig. 3a), indicating that the ECNPs become saturated with the maximum amount of quercetin that can be incorporated at 12–14 wt% and the excess quercetin used in the synthesis simply precipitates out. This hypothesis is supported by the experimental observation that more precipitate is observed after the antisolvent precipitation when larger amounts of quercetin are added to the synthesis. Addition of larger amounts of *p*-coumaric acid than 17 wt% led to the formation of a separate set of micron-sized particles of excess *p*-coumaric acid, as seen by DLS (Fig. S7†). This is why Fig. 3 does not show data points for larger additions of *p*-coumaric acid. Addition of larger amounts of retinol than 50 wt% similarly resulted in the formation of a separate set of much larger particles of excess retinol (Fig. S8†). Interestingly, these maximum loading values of retinol, quercetin, and *p*-coumaric acid into ECNPs are comparable to the synthetic UV filters octinoxate, oxybenzone, and avobenzone, which show maximum loadings of 47 wt%, 14 wt%, and 8 wt% respectively into similarly-sized ECNPs (70–90 nm).^23^ We have previously hypothesised that the discrepancy between loadings between compounds may be a result of the varying solubility of the compound in EC,^23^ which is analogous to the high loadings of SLNs with lyophilic drugs^32^ and polycarbonate NPs with hydrophobic drugs.^33^

The size and stability of the ECNPs as a function of increasing loadings of quercetin and retinol (except for the largest loading of retinol) demonstrated similar behaviour in which the average particle size remained constant (70–76 nm) and absolute zeta potential would increase, indicating increased dispersion stability. This behaviour is similar to the behaviour displayed by the synthetic UV filters oxybenzone, octinoxate, and avobenzone upon encapsulation into ECNPs, where dispersion stability also increased as a function of loading and average particle size remained constant (for loadings below 50 wt%).^23^ However, increasing loadings of *p*-coumaric acid undesirably resulted in a large average particle size increase (70–140 nm) and decreasing absolute zeta potential – indicating decreased dispersion stability. Loading the ECNPs with quercetin and retinol is therefore not limited by decreasing dispersion stability, unlike for *p*-coumaric acid.

Quercetin and retinol are therefore very attractive for the preparation of effective fully-biobased UV-absorbing NPs from EC because of (i) high maximum loading values of quercetin and (particularly) retinol when compared with synthetic UV filters into similarly-sized ECNPs,^23^ and (ii) because higher loadings of quercetin and retinol are not limited by decreasing dispersion stability.

### Retention of biobased photoprotectants inside ECNPs

We also investigated the retention of the three biobased photoprotectants inside the ECNPs. Physical encapsulation of UV filters into nanoparticles is known to be prone to leaching, since the molecules are not covalently bound to the nanoparticle.^13,34^ Although covalent encapsulation of organic UV filters is possible and has been shown to dramatically increase retention,^13^ covalent encapsulation is unrealistic for commercial use because these modified molecules need to be legislatively reapproved. For example, in the U.S. only 3 new UV filters (zinc oxide, avobenzone, and ecamsule) have been approved since 1978.^35^

We found that the biobased photoprotectants quercetin and retinol were effectively retained inside the ECNPs with 64–70% after 72 hours (Table 1, Fig. S11†), where retention values were similar to synthetic UV filters physically encapsulated into nanoparticles developed for sunscreen applications.^13,36,37^ The retention of *p*-coumaric acid could not be measured accurately *via* our spectrophotometric method because the molecule is prone to *cis*–*trans* isomerisation which changes the absorbance.^30^ For these retention experiments the absorbance of 10 mL of the UV absorbing nanoparticles in dialysis tubing was measured at time intervals when sitting in a very large body of water (300 mL). It must be noted that these results represent the retention in the particles when in large bodies of water, as opposed to smaller volumes of water, like in formulations. The retention in the ECNPs in formulations will likely be far greater, potentially allowing for long shelf life of the formulations. These biobased UV-absorbing nanoparticles therefore demonstrate effective retention values, with the additional advantage that if these biobased photoprotectants are released into the environment they are not reported to have such detrimental environmental impacts as their synthetic counterparts to *i.e.* coral reefs.

**Table 1:** Percentage of biobased photoprotectants retained in the ECNPs as a function of time

Sample	Retention (%)		
	24 h	48 h	72 h
Quercetin	86	71	70
Retinol	85	67	64

## Conclusions

In conclusion, we prepared UV-absorbing nanoparticles with an entirely biobased composition *via* the encapsulation of biobased photoprotectants into nanoparticles from ethyl cellulose and zein. The composition of the ECNPs and ZNPs could be easily tuned in order to exhibit uniform, broadband UV spectrum absorbance profiles. We then prepared fully-biobased, transparent coatings with broadband and tunable absorbance by spin coating aqueous dispersions of the UV-absorbing ECNPs. We found that all the biobased photoprotectants could be encapsulated efficiently into the ECNPs to maximum loadings of 31 wt%, 14 wt% and 13 wt% for retinol, quercetin and *p*-coumaric acid respectively, which is comparable to synthetic organic UV filters encapsulated into similarly-sized ECNPs. We found that increasing loadings of quercetin and retinol inside the ECNPs resulted in more favourable behaviour than increasing loadings of *p*-coumaric acid, where the ECNP size remained <100 nm (except for the largest loading of retinol, 31 wt%, where the size increased to 102 nm) and the absolute zeta potential (thus dispersion stability) increased. We also found that the biobased photoprotectants were effectively retained inside the ECNPs at 64–70% after 72 hours, which is comparable to synthetic organic UV filters encapsulated into nanoparticles for sunscreen applications.

Photoprotection technology is pushing towards biobased alternatives for inorganic and synthetic organic UV filters because of their association with many adverse health and environmental effects. Here we develop effective fully-biobased UV-absorbing nanoparticles, representing significant progress towards potentially satisfying these issues, but further studies on the toxicity/environmental impact of plant-based photoprotectants are required.

## Conflicts of interest

There are no conflicts to declare.

## Supplementary Material

RA-008-C8RA02674B-s001
