# Supplementary material for: Fully-biobased UV-absorbing nanoparticles from ethyl cellulose and zein for environmentally friendly photoprotection
Source: RSC Adv. 2018 Jul 12;8(44):25104–11. doi: 10.1039/c8ra02674b (PMC9082358; doi:10.1039/c8ra02674b)
Supplement: RA-008-C8RA02674B-s001 [file RA-008-C8RA02674B-s001.pdf]

Electronic Supplementary Material (ESI) for RSC Advances.
This journal is © The Royal Society of Chemistry 2018

Supporting Information

Fully-Biobased UV-Absorbing Nanoparticles from Ethyl Cellulose and Zein for Environmentally Friendly Photoprotection

Douglas R. Hayden[a‡], Heleen V. M. Kibbelaar[a‡], Arnout Imhof[a*], Krassimir P. Velikov[a, b, c]*

[a] Soft Condensed Matter, Debye Institute for Nanomaterials Science, Utrecht University, Princetonplein 1, 3584 CC, Utrecht, the Netherlands

[b] Unilever R&D Vlaardingen, Olivier van Noortlaan 120, 3133 AT Vlaardingen, the Netherlands

[c] Institute of Physics, University of Amsterdam, Science Park 904, 1098 XH Amsterdam, The Netherlands

Corresponding Authors

*Email: d.r.hayden@uu.nl, a.imhof@uu.nl

1. Preparation of Fully-Biobased ECNPs and ZNPs with Broadband UV Absorbance

The ECNPs and ZNPs with quercetin, p-coumaric acid, and retinol encapsulated together in a 7:1.5:1.5 ratio respectively (from Figure 1) show average particle size measurements of 72 nm (for ECNPs, PDI = 26.6 nm) and 76 nm (for ZNPs, PDI = 30.2 nm) as determined by DLS (Figure S1).

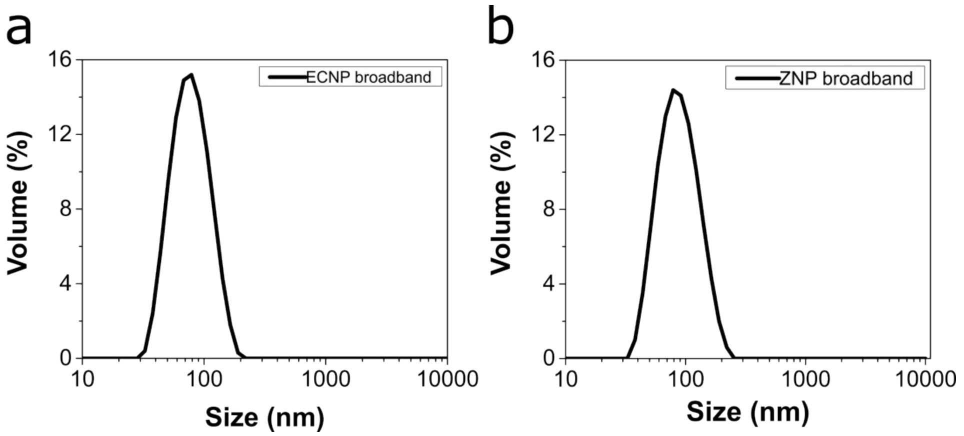

Figure S1. Size distributions as determined by DLS for the (a) ECNPs and (b) ZNPs with quercetin, retinol and p-coumaric acid encapsulated in the ratio 7:1.5:1.5.

We found that we could simply tune the amount of biobased photoprotectant encapsulated into the ECNPs by the addition of varying ratios of photoprotectant (absorbance profiles shown in Figure S2a) in the ethanol solution. We found that a 1:1:1 (3.3 wt% ratio for each of retinol, p-coumaric acid and quercetin) resulted in an absorbance profile which was lacking in UVA coverage (Figure S2b). We always kept the total loading below 10 wt.% because ECNPs are known to encapsulate organic UV filters efficiently to this weight percentage[1], and we wanted to stay under this value so that the encapsulation efficiency remained high. This meant that the

absorbance profiles from the ECNPs were reproducible. We found that a ratio of 7:1.5:1.5 provided uniform UV spectrum coverage.

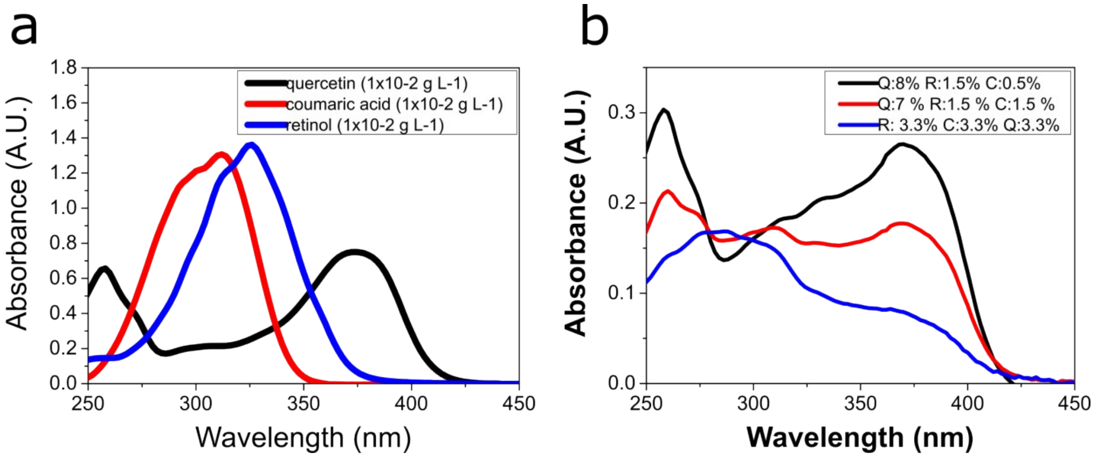

Figure S2. (a) Absorbance profiles of quercetin, coumaric acid, and retinol all measured at equal concentrations of photoprotectant in ethanol (1×10^{-2} g L^{-1}). (b) Absorbance measurements of ECNPs with the three UV filters encapsulated in varying ratios in which the total loading was kept under 10 wt.%. All measured at equal ECNP concentrations (5×10^{-2} g L^{-1}).

We also investigated loading the ECNPs with greater than 10 wt% material in order to prepare thinner coatings with higher absorbance. Spin coating multiple layers can sometimes result in non-uniformity and this can be avoided by preparing thinner coatings with already high absorbance. We found that this was possible by simply dissolving more material in the initial ethanol solution, and that the ECNPs showed a much greater absorbance at the same concentration of particles (Figure S3 vs. Figure S2b). However, we found that the absorbance profile was not easy to predict, probably because not all the biobased photoprotectants which were added to the synthesis ended up encapsulated. For example in Figure S3 we see that a 10:3:3 ratio [Q:R:C] results in higher absorbance below 350 nm than a 15:15:4 ratio. This is likely because the ECNPs are unable to encapsulate material after a certain maximum point,

which is consistent with our "Maximum Particle Loading" results in Figure 3a where we find that quercetin and p-coumaric can only be encapsulated to a maximum of between 12-14 wt.%. Also, the final ratio of the UV filters encapsulated may then be a result of factors other than just the maximum loading for each UV filter, including potential interactions between UV filter molecules with each other (and also with ethyl cellulose) when confined into a small space.

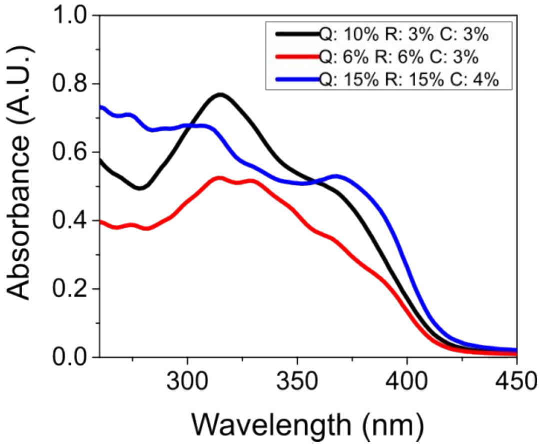

Figure S3. Absorbance profiles of ECNPs with the three biobased photoprotectants encapsulated in varying ratios. All measured at equal ECNP concentrations (5 x 10^{-2} g L^{-1}).

2. Preparation of Coatings from Full-Biobased UV-absorbing ECNPs

In Figure 2 we showed the preparation of spin coatings of the ECNPs with quercetin, p-coumaric acid and retinol encapsulated in the ratio 7:1.5:1.5. We also prepared spin coatings from the ECNPs with greater amounts of encapsulated material, such as those from Figure S3 (Figure S4). We found that spin coating the ECNPs with a 15:15:4 ratio of encapsulated UV filters (quercetin, p-coumaric acid, and retinol) gave thinner coatings with greater absorbance than an equal concentration (30 g L^{-1}) of the ECNPs with less encapsulated material (Figure S4 vs. Figure 2b in main article). The spin coatings were again prepared onto plasma-cleaned,

circular glass microscope cover slips at 1800 rpm for 1 minute. This way, highly transparent and thin coatings high absorbance could be prepared.

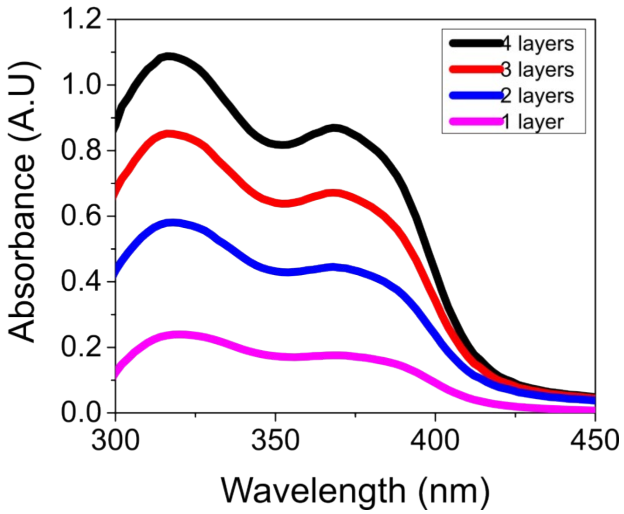

Figure S4. Absorbance measurements of the ECNP coating prepared from the ECNPs with a 15:15:4 ratio of encapsulated quercetin, p-coumaric acid, and retinol. The absorbance is shown for 4 successive spin coated layers. Wavelengths lower than λ = 290 nm are not shown as they are absorbed by the glass coverslip.

In Figure S5 we show a SEM image of the cross section of a glass slide with a 4 layer coating. This SEM image was used to determine the coating thickness (373 ± 17 nm) on the glass slide by taking the average of 10 measurements at different areas along the cross section.

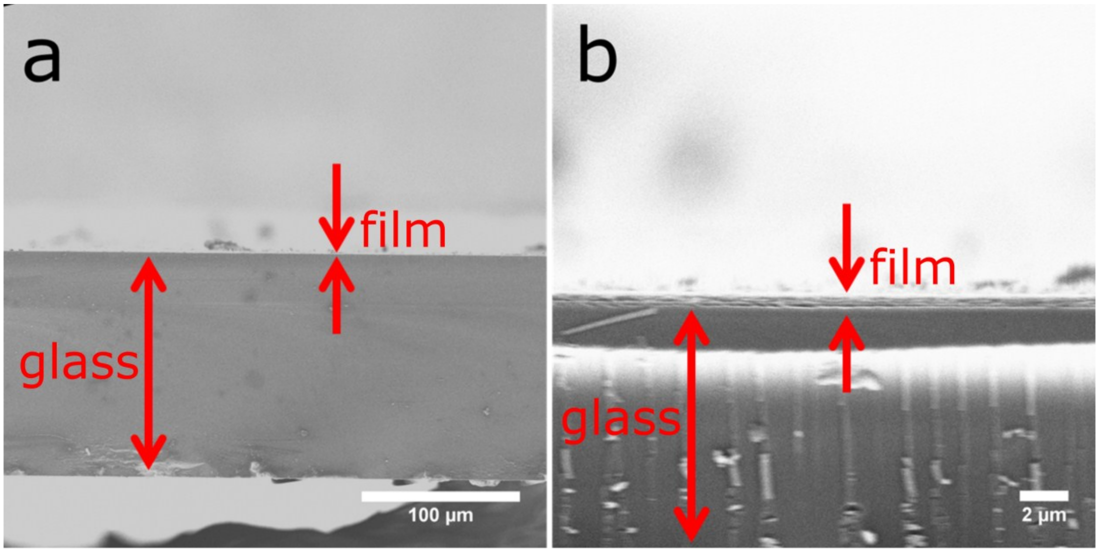

Figure S5. SEM images of the cross section of a glass slide covered with a 4 layer coating of fully-biobased UV-absorbing ECNPs, (a) at low magnification and (b) at high magnification where the thin coating is visible.

In Figure S6 we show a SEM image of the coating surface after irradiation by UV light. We can see that there is no significant change in size or shape of the ECNPs as a result of the irradiation with UV light.

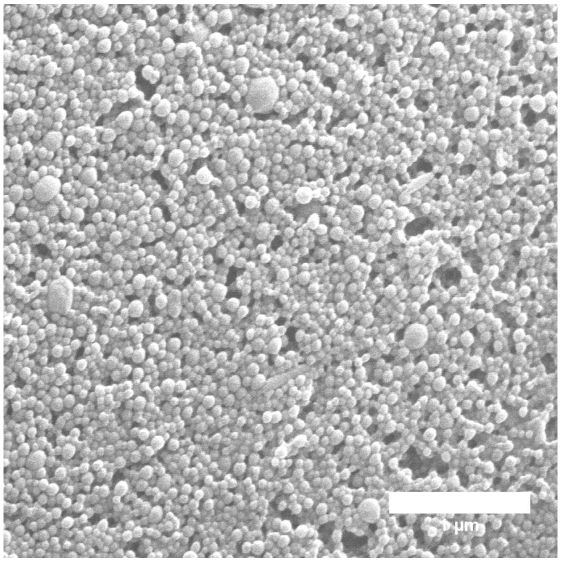

Figure S6. SEM image showing the coating surface after UV irradiation. Scale bar 1 µm.

3. Investigation into the Encapsulation of Individual Biobased Photoprotectants into ECNPs

Increasing loadings of p-coumaric acid into the ECNPs resulted in a considerable change in the average ECNP size (Figure S7). Moreover, we found that the addition of p-coumaric acid greater than 17 wt% resulted in the formation of a second set of micron-sized particles. This larger set of particles is likely pure p-coumaric acid or p-coumaric acid with some EC at the interface: the same micron-sized particles are formed when the antisolvent precipitation is performed with pure p-coumaric acid and no EC (Figure S7f). This observation has previously been reported for the loading of large amounts of synthetic organic UV filters into ECNPs of similar size (<100 nm)[1].

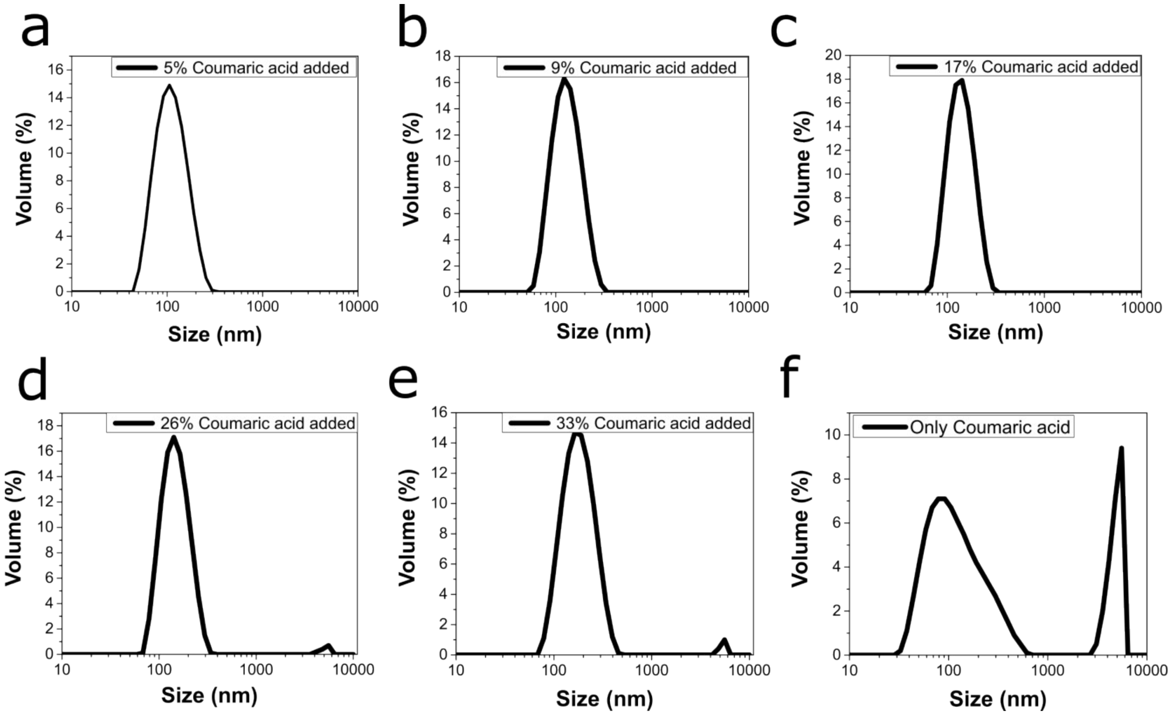

Figure S7. Size distributions as measured by DLS for the ECNP dispersions with encapsulated p-coumaric acid in Figure 3.

Retinol could be loaded into the ECNPs very efficiently, up to 31 wt%. The average particle size did not change upon increasing loadings (70-76 nm) except for the largest loading in which the size increased slightly to 102 nm (Figure S8). The increase in size probably arises because of with the greater amount of material in the particle, and this phenomenon has been seen previously for ECNPs with high loadings (47 wt%) of the UV filter octinoxate[1]. We found that the addition of large amounts of retinol (66 wt%) resulted in the formation of much larger particles along with the ECNPs. Similarly as to the case with p-coumaric acid, these larger particles are likely pure retinol or retinol stabilised by some EC at the interface: large particles are also formed upon performing the antisolvent precipitation of retinol alone (Figure S8h).

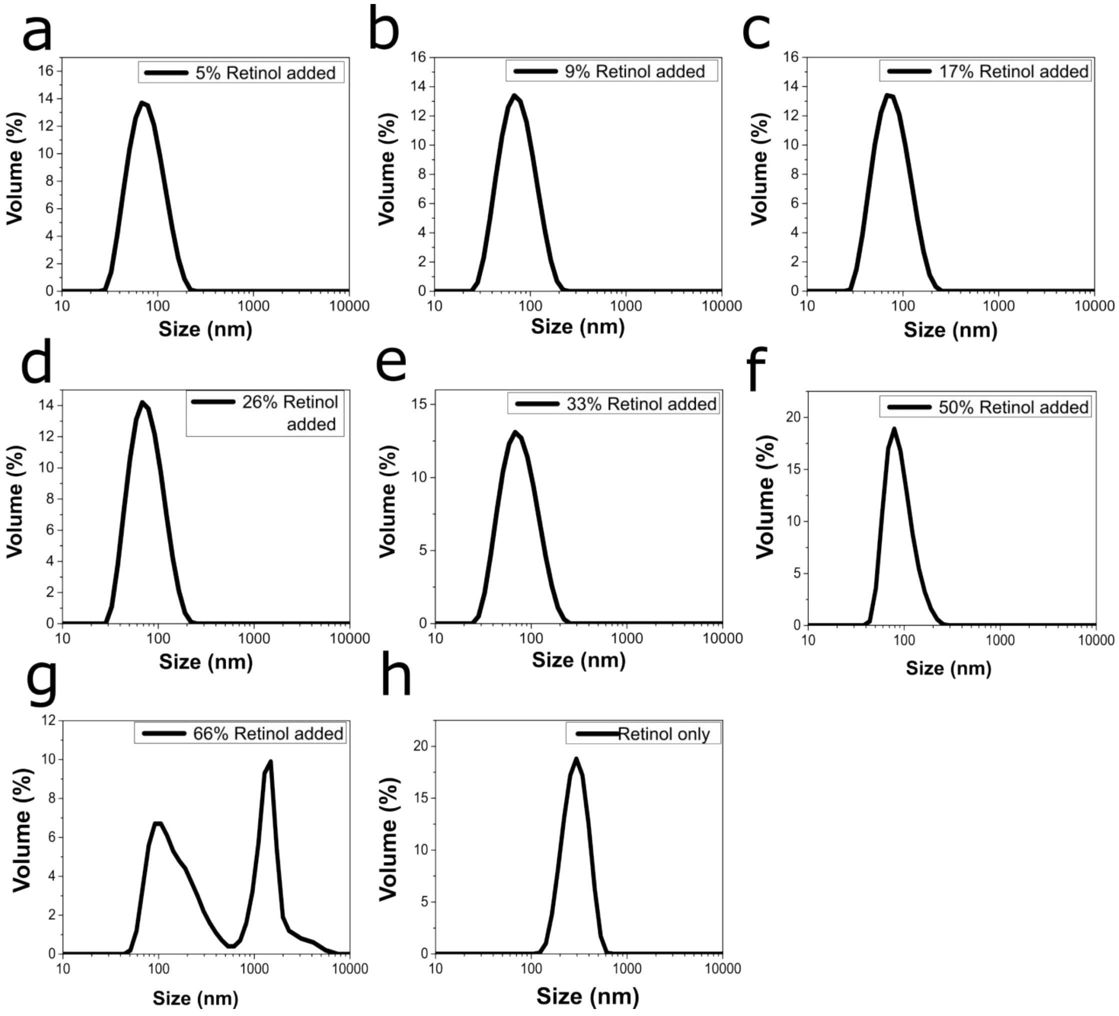

Figure S8. Size distributions as measured by DLS for the ECNP dispersions with encapsulated retinol in Figure 3.

Increasing loadings of quercetin into the ECNPs resulted in no change in the average particle size (Figure S9). We hypothesise that this is because the loadings are still relatively low.

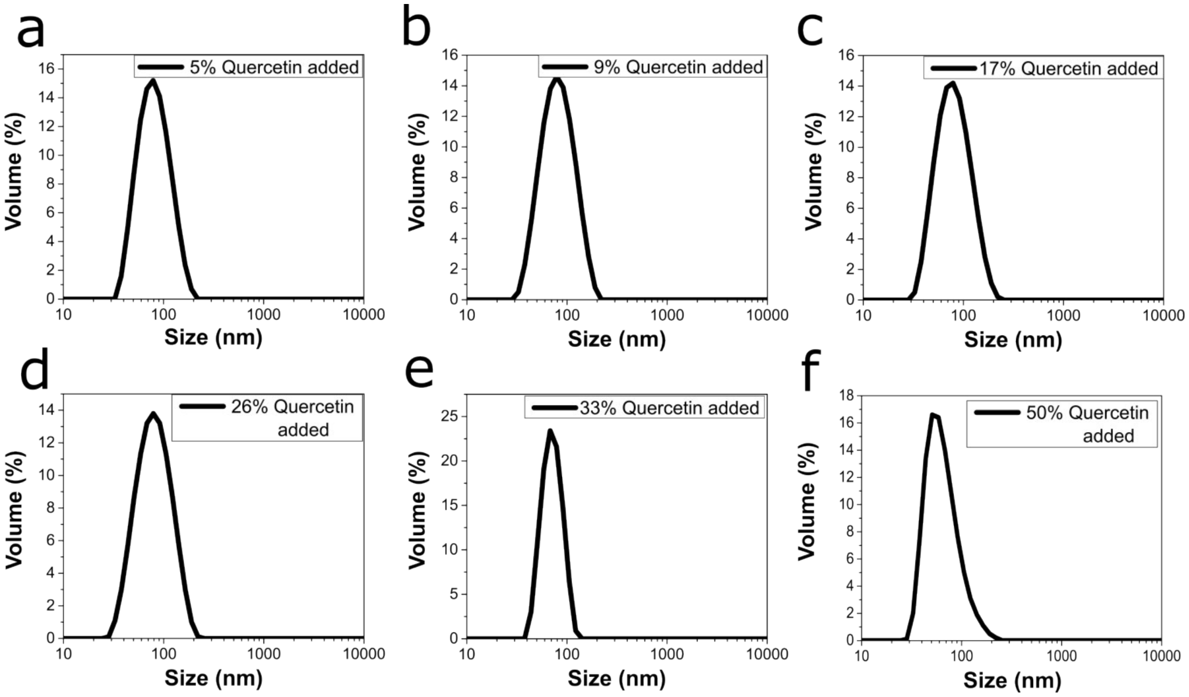

Figure S9. Size distributions as measured by DLS for the ECNP dispersions with encapsulated quercetin in Figure 3.

The raw data for Figure 3 can be found in Table S1 below.

Table S1. Raw data for Figure 3.

Sample	Loading	error	z-average size (nm)	Error (nm)	PDI width (nm)	Zeta pot. (mV)	error
5 wt% Quercetin	3.8	1.6	76	0.1	28	-27.9	0.1
9 wt% Quercetin	7.1	0.7	75	0.1	30	-33	0.5
17 wt% Quercetin	12.0	1.4	76	1.1	32	-36.5	0.1
26 wt% Quercetin	14.0	0.8	77	0.7	31	-32	0.5
33 wt% Quercetin	13.0	2.5	77	0.5	34	-33.5	0.1
50 wt% Quercetin	8.6	2.6	82	0.9	33	-35	0.4
5 wt% Retinol added	2.7	0.1	68	0.1	27	-26.0	0.5
9 wt% Retinol added	7.1	0.3	69	0.1	29	-34.6	0.5
17 wt% Retinol added	12.6	2.8	71	0.5	29	-38.2	1
26 wt% Retinol added	15.9	0.1	72	0.5	31	-36.5	0.5
33 wt% Retinol added	20.2	3.9	73	0.1	34	-37	1
50 wt% Retinol	30.9	3.5	102	0.5	46	-38.8	0.5
5 wt% p-Coumaric	1.9	0.2	93	0.5	32	-24	0.1
9 wt% p-Coumaric	6.9	0.5	120	0.1	31	-16	0.5
17 wt% p-Coumaric	12.8	0.9	131	0.5	51	-12	0.5
Empty ECNPs	0	0	71	0.4	24	-26	1.8

The loadings from Figure 3 were determined by a spectroscopic method, where the absorbance of a known concentration of aqueous ECNPs containing encapsulated photoprotectant at the peak of the spectrum was compared with a calibration curve prepared from a series of known concentrations of photoprotectant dissolved in ethanol. Here we make the assumption that the contribution of particle scattering to absorbance is minimal. This assumption is supported by Figure S10 which shows the absorbance shown by the ECNPs alone is very small compared to the absorbance profile from the series which were used to determine the loadings, when measured at similar concentrations (actual concentrations shown in Figure S10). The potential inaccuracy of the final loading which arises as a result of this assumption is smaller

than 0.6 wt% for the loadings of p-coumaric acid, which is the photoprotectant for which the particle scattering contributes most to the peak absorbance (because the peak maximum of p-coumaric acid is at the shortest wavelength λ=310 nm of all the photoprotectants and the absorbance due to particle scattering is greatest at lower wavelengths). The potential inaccuracy due to this assumption is much lower for the other photoprotectants (<0.1 wt%). We therefore consider the potential inaccuracy due to this assumption negligible for all the photoprotectants. We determined the loadings in this way and not by drying the particles and re-dissolving in another solvent because retinol is unstable upon direct contact with air and both retinol and p-coumaric acid are unstable as a result of elevated temperatures which would be required to dry the particles[2,3]. This way, we could determine the loadings for all the UV filters using an identical technique and without damaging the photoprotectants (and therefore reducing the chance of inaccurate results).

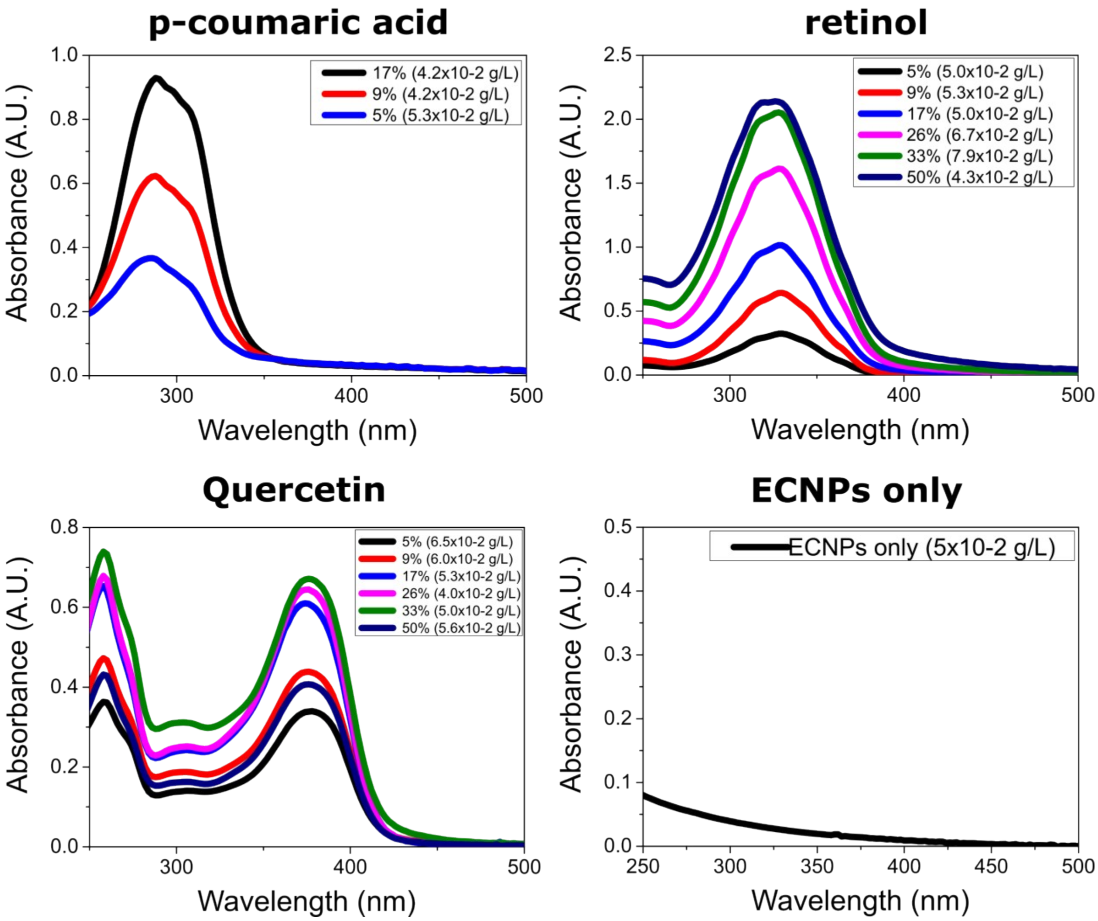

Figure S10. Absorbance measurements of ECNPs with encapsulated p-coumaric acid, retinol, quercetin, and ECNPs with nothing encapsulated. These absorbance measurements were used to determine the particle loadings. The absorbance of the ECNPs due to scattering is very small compared to the absorbance profiles for the used to determine the particle loadings. The legends show the amount of photoprotectant added to the synthesis (akin to the x-axis on Figure 3a) as well as the concentration of the ECNP dispersions at which they were measured by spectrophotometry.

4. Retention of the Biobased Photoprotectants inside the ECNPs

In Figure S11 we show the absorbance measurements of 10 mL aliquots of ECNPs containing retinol and quercetin (both dispersions 5 g/L) in dialysis tubing measured after 0, 1, 2, and 3 days. Percentage retention is in Table 1.

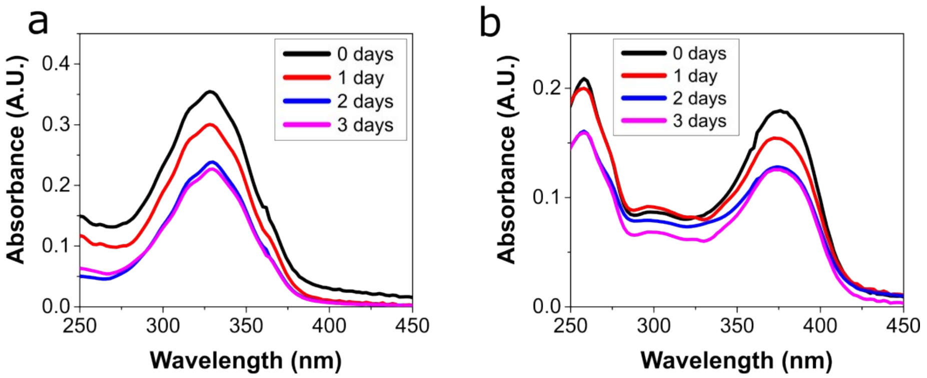

Figure S11. Absorbance measurements showing the decrease in absorbance due to the release of ECNPs containing (a) 3 wt% of retinol and (b) 4 wt% quercetin from dialysis tubing into the surrounding water medium over a period of 3 days.

REFERENCES

1 D. R. Hayden, H. Kibbelaar, A. Imhof and K. P. Velikov, *ChemNanoMat*, 2018, **4**, 301–308.

2 R. Kort, H. Vonk, X. Xu, W. D. Hoff, W. Crielaard and K. J. Hellingwerf, *FEBS Lett*, 1996, **382**, 73–78.

3 J. K. Rutz, R. C. Zambiazi, C. D. Borges, F. D. Krumreich, S. R. Da Luz, N. Hartwig and C. G. Da Rosa, *Carbohydr. Polym.*, 2013, **98**, 1256–1265.
